# Mixed Reality–Based Slit Lamp for Ophthalmic Examination and Telemedicine: Technological Development and Validation Study

**DOI:** 10.2196/93513

**Published:** 2026-05-14

**Authors:** Rui Zhou, Wei-Chiang Lin, Byron L Lam, Rong Wen, Noble Amadi, Shuliang Jiao

**Affiliations:** 1 Department of Biomedical Engineering, Florida International University, 10555 W Flagler St, EC-2610, Miami, FL, 33174, United States, 1 3053484984; 2 Bascom Palmer Eye Institute, University of Miami Miller School of Medicine, Miami, FL, United States

**Keywords:** telemedicine, teleophthalmology, mixed reality, slit-lamp microscopy, depth perception, wearable, remote consultation

## Abstract

**Background:**

The slit-lamp biomicroscope is a fundamental diagnostic tool in ophthalmology for detailed examination of the eye. Current camera-equipped digital slit lamps were designed with a single optical channel, which results in the loss of depth information. Without that information, it can be challenging to visualize subtle anatomical variations in teleophthalmology applications and perform procedures guided by the digital view.

**Objective:**

This study aimed to present a feasibility study of the mixed reality (MR)–based slit lamp (MR-SLP) capable of transmitting real-time stereoscopic views of the slit lamp to local and remote MR headsets, enabling stereoscopic teleophthalmology.

**Methods:**

A prototype MR-SLP was built by integrating a calibrated stereoscopic camera pair on the left and right viewing channels of a conventional slit lamp and a real-time streaming network. The stereoscopic diagnostic images were transmitted to multiple MR headsets through the streaming network at 1080p and 30 frames per second (fps). The spatial resolution of the system was quantified using a US Air Force 1951 resolution target (Edmund Optics Inc). The 3D spatial accuracy and coordination were evaluated quantitatively by performing a tube-threading test. Five participants (mean age 42.2, SD 16.5 years) with normal visual function, best-corrected visual acuity of 20/20 or better, and a minimum stereoacuity of approximately 40 arc seconds participated in the tube-threading test. Teleophthalmology capability was assessed through real-time streaming across multiple remote sites at Florida International University and Bascom Palmer Eye Institute.

**Results:**

The measured spatial resolution reached 102 line pairs/mm at 25× optical magnification. The tube-threading task was performed under 4 conditions. Task performance differed significantly between nonstereoscopic (2D) and direct eyepiece views (*P*=.03, Kruskal-Wallis test), but not between stereoscopic (3D) MR and direct views (*P*>.05, Kruskal-Wallis test). In the real-time remote streaming tests across multiple sites, the system achieved stable, low-latency transmission with an average round-trip time below 40 milliseconds. Participating ophthalmologists reported user experience and image quality comparable to traditional slit lamps.

**Conclusions:**

The MR-SLP can provide real-time stereoscopic slit-lamp examination images and videos through a broadcasting network to local and remote locations. The spatial resolution and visuomotor performance are comparable to direct viewing through the eyepieces of a traditional slit lamp. This study demonstrated the feasibility of the MR-SLP for high-quality stereoscopic teleophthalmology.

## Introduction

The slit-lamp biomicroscope is a fundamental diagnostic tool in ophthalmology for detailed examination of both the anterior and posterior segments of the eye [1] with stereoscopic visualization [2]. Traditional slit lamps require the operator to view through the eyepieces to obtain a stereoscopic perspective. This necessity means the operator must stay close to the patient, which has become more concerning since the COVID-19 pandemic, given that close contact increases the risk of transmitting infectious diseases [3,4]. Modern slit lamps frequently come with a camera, whether dedicated or integrated into a smartphone, allowing them to capture images and videos of the eye during examinations. This feature is especially beneficial for data collection, telemedicine, and education, as captured images and videos can be viewed on a display, stored electronically, and shared remotely [5-7]. Current camera-equipped slit lamps often use a single-channel design, resulting in the loss of stereoscopic information and, consequently, depth perception during image and video capture. Without depth perception, it can be challenging to visualize subtle anatomical variations and perform procedures guided by the slit-lamp view [8].

Mixed reality (MR) technology enables interactive experiences that blend both real and virtual environments by using a head-mounted display (HMD). A HMD is a wearable device designed to fit comfortably over the user’s head, equipped with a stereo camera and individual screens for each eye. Additional sensors are incorporated to accurately track head and eye movements. The MR headset’s see-through function allows users to view the real world with depth perception. By seamlessly blending digital content with the real world, MR facilitates intuitive interaction and manipulation of virtual objects within the user’s actual environment. These advancements make MR a promising tool for applications such as 3D medical data visualization and telemedicine [9,10].

To address the limitations of conventional slit-lamp examinations in remote collaboration and stereoscopic information sharing, we developed the MR-based slit lamp (MR-SLP) that integrates MR technology with a traditional slit lamp. The MR-SLP enables local operators to interact naturally with patients using its see-through function while simultaneously visualizing a stereoscopic diagnostic view; it also allows real-time streaming of the view to remote physicians wearing headsets for immersive participation. This functionality supports collaborative decision-making, as remote specialists can actively guide local operators during examinations, and enables recording of stereoscopic diagnostic images and videos for documentation and education. In this study, we aim to develop and evaluate the MR-SLP system and assess its capability for real-time stereoscopic imaging, remote visualization, and collaborative ophthalmic examination and telemedicine.

## Methods

### Ethical Considerations

The study was conducted in accordance with the tenets of the Declaration of Helsinki and was approved by the institutional review board (IRB) of Florida International University (IRB-19‐0112-CR02 and IRB-26‐0004). Written informed consent was obtained from all participants prior to enrollment. All data were deidentified before analysis, and no personally identifiable information or protected health information was collected or stored by the MR-SLP system.

### System Design

The MR-SLP system developed in this study was built on a commercial slit lamp (XCEL250, Recheit Inc). Two board-level USB video cameras (ELP-USB16MP01-L75, 1/2.8-inch sensor, maximum resolution: 4656×3496 pixels) formed a stereoscopic pair and were calibrated before mounted on the left and right eyepieces using custom-designed adapters. The adapters allow fine-tuning of the cameras’ positions to ensure accurate alignment of their optical axes with the eyepieces. A schematic of the system is shown in Figure 1.

**Figure 1.**
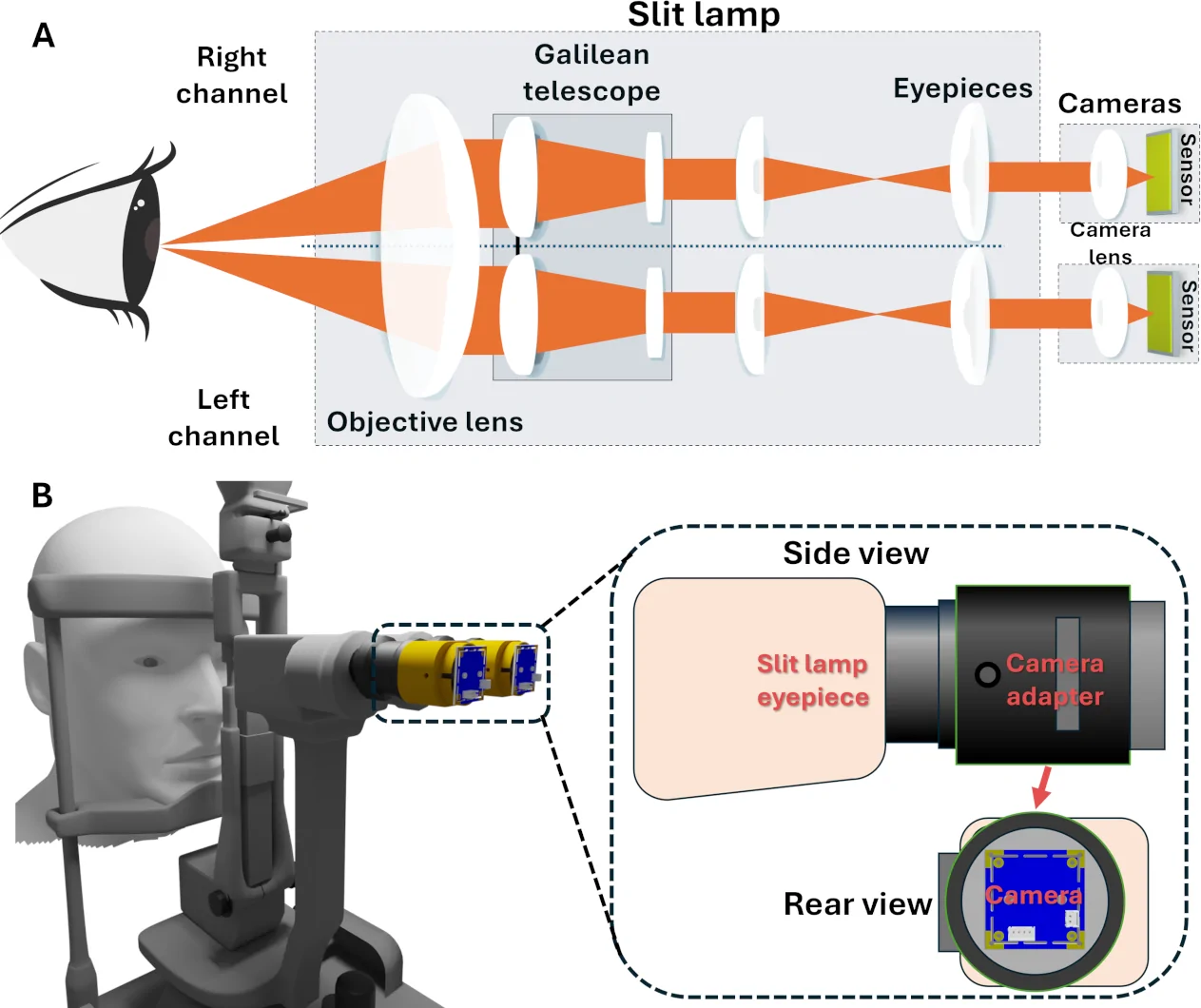
Design of the mixed reality–based slit-lamp (MR-SLP) system. (A) Schematic of the optical path of the MR-SLP. Light reflected by the examined area enters the front objective lens, then proceeds through the Galilean telescope assembly and the eyepieces before reaching the camera lens and being detected by imaging sensors. (B) Adaptation of a traditional slit lamp for MR-SLP functionality. Two cameras are mounted on the slit-lamp eyepieces using adapters, allowing for stereoscopic ocular imaging.

The videos captured by the 2 cameras were first streamed via USB to a local computer, where they were processed in real time using Open Broadcaster Software Studio (64-bit, version 31.0), an open-source video streaming application. For the video processing pipeline in Open Broadcaster Software Studio, we used the NVIDIA NVENC H.264 hardware encoder to achieve efficient, low-latency streaming. The encoder settings were specifically tuned for real-time performance. Key parameters included rate control set to constant bitrate, bitrate at 10 Mbps, keyframe interval set to 0 seconds for automatic management, and preset configured to “fastest” with “ultra low latency” tuning. The stream used the “baseline” profile to maximize compatibility and minimize processing overhead. The processed videos were formatted into side-by-side 3D mode (Figure 2A), a widely used format for conveying stereoscopic information in image processing [11]. Subsequently, the side-by-side 3D video was transmitted to local and remote Web Real-Time Communication (WebRTC) servers using the WebRTC HTTP ingestion protocol, with an output resolution of either 1080p or 720p. For local streaming, a free WebRTC server (OSSRS/SRS v6.0 from Docker Hub) was deployed on a PC, while remote streaming was facilitated by a commercial WebRTC service provided by dolby.io over the internet. The processed streams were broadcasted wirelessly to MR headsets using the WebRTC HTTP egress protocol. The headsets (Meta Quest 3; Meta Platforms Inc) then rendered the stream into a stereoscopic view with their built-in video player for immersive visualization (Figure 2B). An image in red-cyan stereoscopic format is provided (Figure 2C) as an example, which can be viewed using red-cyan anaglyph 3D glasses. A diagram of the broadcasting architecture is shown in Figure 2D.

**Figure 2.**
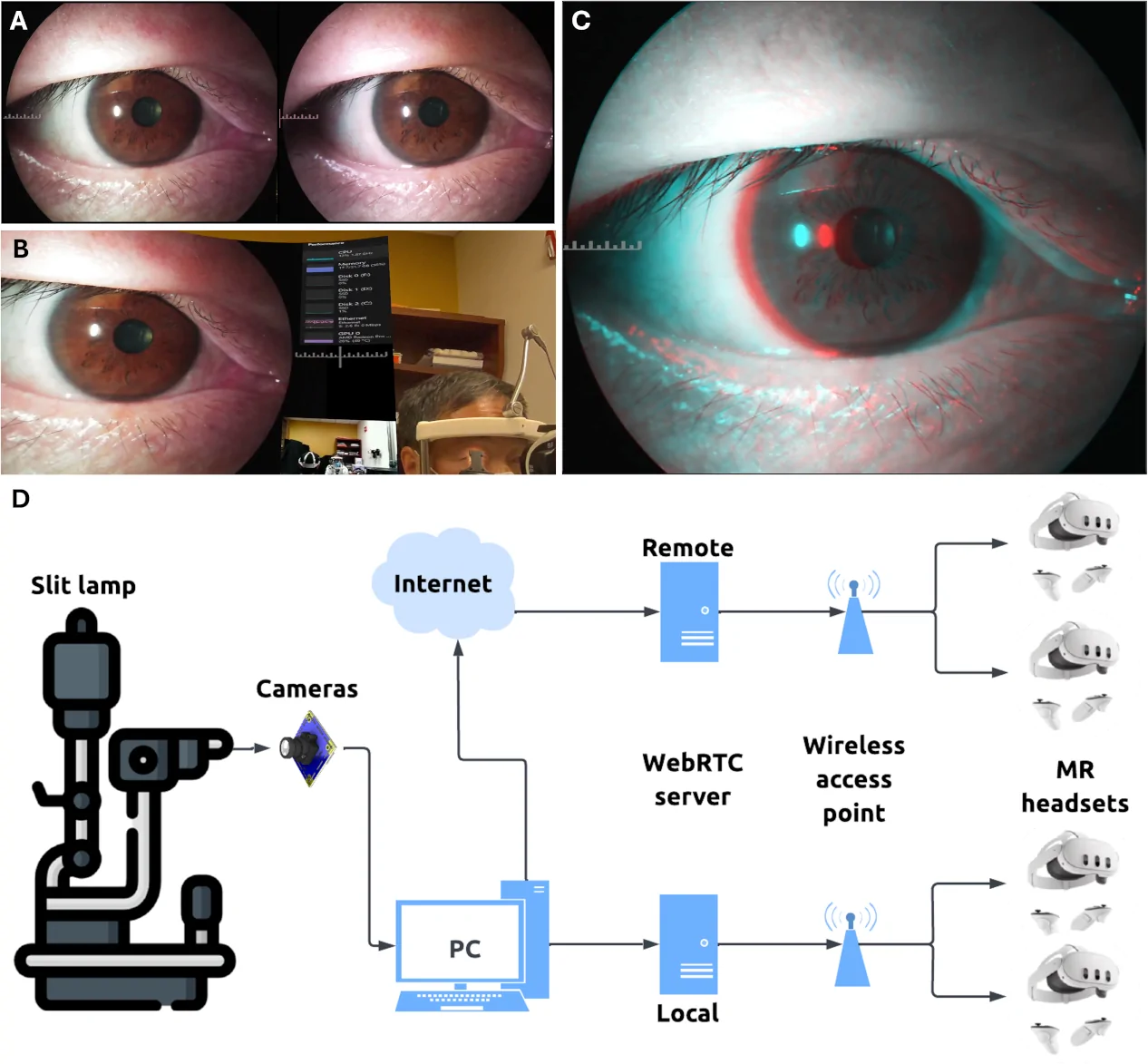
Imaging procedures from camera to the head-mounted display (HMD)–rendered display. (A) Example images of a participant’s eye captured with the mixed reality (MR)–based slit-lamp (MR-SLP) cameras in the side-by-side 3D (SBS) format. (B) The view of the local operator through their MR headset. The embedded window on the left displays the stereoscopic view (rendered from the SBS video) of the participant’s eye. The participant and the environment are seen with the see-through function of the MR headset. (C) An image in red-cyan stereoscopic format demonstrates the 3D view as an example that can be observed using red-cyan anaglyph 3D glasses. (D) Broadcasting system architecture. The videos captured by the cameras on a slit lamp are processed by a local PC and transmitted wirelessly to local and remote MR headsets via Web Real-Time Communication (WebRTC) servers.

### Spatial Resolution Evaluation

A US Air Force 1951 resolution target (Edmund Optics Inc) was used to evaluate the spatial resolution of the MR-SLP. The target was placed at the imaging plane of the MR-SLP and imaged at 25× optical magnification. Raw images of the resolution target were captured using the MR-SLP’s USB cameras with the built-in camera app on Microsoft Windows 11, at a resolution of 4656 × 3496 pixels, which is the cameras’ maximum resolution. Images displayed in the MR headset were captured through real-time video streaming, using the built-in snapshot tool of the Meta Quest 3 at a resolution of 1080p.

Intensity profiles were generated across the line pairs for selected group–element combinations from the acquired resolution target images using MATLAB (MathWorks Inc). The minimum resolvable line pairs were determined according to the Rayleigh criterion, wherein the central maximum of one pattern coincides with the first minimum of another. Additionally, a subjective evaluation was conducted in which participants compared the minimum readable line pairs observed directly through the eyepieces with those visible in the images captured via camera.

### Tube-Threading Test for Evaluating Visuomotor Performance With the MR-SLP

To assess the visuomotor performance of the MR-SLP, a tube-threading test was designed based on the concept of a bead-threading task [12,13], a well-known method for estimating fine visuomotor performance that was critically affected by depth perception. This tube-threading test consisted of 2 sequential tasks. In task 1, the participant was asked to pick up each of the 10 tubes from a container using a tweezer, place the tube at the focal plane of the slit lamp to be seen clearly, and then return the tube to the container. In task 2, the participant was asked to pick up each tube, thread a wire into it under the visual guidance of the slit lamp, and return the tube to the container. The participants were required to complete 2 tasks 5 times under each designed condition shown in Table 1. High-precision tweezers (PL-30; Fisher Scientific) primarily for microscopy applications were used to grab the tubes. The tubes used in this test were made from micropipette tips. Their outer diameter ranged from 0.8 mm to 1.6 mm, and their inner diameter ranged from 0.4 mm to 1.2 mm. The diameter of the wire used in this test was 0.2 mm. The small size of these tubes and the wire, combined with 10× optical magnification of the slit lamp, required precise depth perception to complete the designed tasks.

Five individuals aged between 21 and 61 years (mean 42.2, SD 16.5 years) were recruited for this test. All participants provided written informed consent to participate in the study. Each participant had normal visual function, with a best-corrected visual acuity of 20/20 or better and a minimum stereoacuity of approximately 40 arc seconds, assessed using random-dot stereograms [14]. All participants did not have any history of ophthalmologic surgery, ocular motility disorders, known fine motor impairments, or other physical impairments that could interfere with the task performance. All participants completed a short acclimation session before the main test. This included (1) adjusting the eyepieces and MR headset to achieve a full field of view at test magnification, (2) finding a comfortable position so that their body and hands were stable and at ease while performing the tube-threading test, and (3) practicing the tube-threading task until they felt comfortable.

**Table 1.** Test conditions used in the tube-threading evaluation.

Condition ID (c)	Conditions	Description
1	MR-SLP^a^, 2D, 60 fps^b^	Nonstereoscopic stream^c^, output resolution 720p, frame rate 60 fps, with MR^d^ headset.
2	MR-SLP, 3D, 30 fps	Stereoscopic stream, output resolution 720p, frame rate 30 fps, with MR headset.
3	MR-SLP, 3D, 60 fps	Stereoscopic stream, output resolution 720p, frame rate 60 fps, with MR headset.
4	Conventional slit lamp	Direct view through the eyepieces of the slit lamp, without MR headset.

a MR-SLP: mixed reality–based slit lamp.

b fps: frames per second.

c Nonstereoscopic stream was created by converting the video from a single mixed reality–based slit-lamp camera into the side-by-side 3D format.

d MR: mixed reality.

The time taken for each task under each condition was individually recorded, denoted as $T_{task1}\left(p,c,i\right)$ and $T_{task2}\left(p,c,i\right)$, where *p* is the participant number, *c* is the condition ID, and *i* is the repeat.

From a single repeat, the time to thread the tube $T_{thr}\left(p,c,i\right)$was calculated as


(1) $$T_{thr}\left(p,c,i\right)=T_{task2}\left(p,c,i\right)-T_{task1}\left(p,c,i\right)$$

The mean of $T_{task1}\left(p,c,i\right)$ and $T_{thr}\left(p,c,i\right)$ from the 5 repeats of a given participant under the same condition were calculated as


(2) $$\begin{array}{l} {\overset{-}{T}}_{task1}\left(p,\;c\right)=\frac{1}{5}\sum_{i=1}^{5}T_{task1}\left(p,\;c,i\right),\\ {\overset{-}{T}}_{thr}\left(p,\;c\right)=\frac{1}{5}\sum_{i=1}^{5}T_{thr}\left(p,\;c,i\right) \end{array}$$

and $T_{task2}\left(p,c,i\right)$ taken for each task under each condition was individually recorded. ${\overset{-}{T}}_{task1}\left(p,c\right)$ and ${\overset{-}{T}}_{thr}\left(p,c\right)$ for all 4 conditions were collectively analyzed using statistical methods to identify the effects of the test conditions on participants’ performance.

### Telemedicine Assessment

A telemedicine assessment was conducted to evaluate the streaming quality, remote usability, and applications of the MR-SLP. Five participants were assigned to different roles across 3 locations in Miami. The local site was at the main campus of Florida International University, where an ophthalmologist with around 10 years of clinical experience operated the MR-SLP and performed test examinations on a volunteer with normal visual function. Stereoscopic slit lamp video was captured and streamed in real time at an output resolution of 1080p. Simultaneously, a second ophthalmologist with more than 20 years of clinical experience watched the stereoscopic video from the MR-SLP using an MR headset at the first remote site, Bascom Palmer Eye Institute at the University of Miami Miller School of Medicine. He communicated in real-time with the MR-SLP operator at the local site, providing expert guidance and diagnostic opinions. In addition, a senior scientist at the first remote site also observed the examination procedure through an MR headset for technical assistance. Finally, a senior scientist at the second remote site, the Florida International University Engineering Center, observed the process using an MR headset. All local and remote participants verbally assessed the system’s technical performance, including image quality and streaming stability. The latency, particularly the round-trip time, was provided by the remote WebRTC platform. Real-time audio communication between the local operator and remote participants was enabled using Zoom (Zoom Communications, Inc).

### Data Analysis

Statistical analyses were performed using R (version 4.5.1; R Foundation for Statistical Computing). The normality of the tube-threading task completion times was assessed using the Shapiro-Wilk test. Given the nonnormal distribution of the data, the nonparametric Kruskal-Wallis test was used to compare conditions, followed by post hoc analysis using the Dunn test with Bonferroni correction. Statistical significance was set at *P*<.05.

## Results

### Image Quality of the MR-SLP: Spatial Resolution

Our objective analysis revealed that both the MR-SLP cameras and MR headset were able to resolve element 5 in group 6 of the resolution target, corresponding to a spatial resolution of 102 line pairs/mm with a line width of approximately 9.8 µm (Figure 3). Additionally, we conducted a subjective evaluation with 3 participants who had a best-corrected visual acuity of 20/20 or better. These participants noted that, although they could discern element 5 in group 6 through the MR headset, they were able to identify up to element 6 in group 6 (114 line pairs/mm) when viewing directly through the slit-lamp eyepieces. All participants noted that the lines appeared slightly clearer when viewed through the traditional slit lamp compared with the MR headset display. This subjective difference may indicate that the fidelity of the current digital imaging pathway, which includes the camera and display, may not match that of direct optical viewing with the human eye.

**Figure 3.**
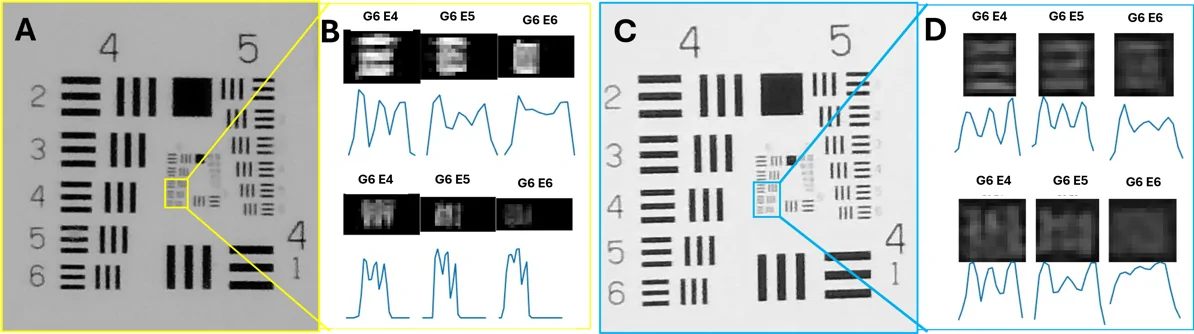
Resolution target imaging. (A) Image of the line pairs (lps) from groups 4 to 6 for the resolution target captured directly by the mixed reality (MR)–based slit-lamp camera. (B) The upper panel shows the intensity profiles across the horizontal lps of elements 4 to 6 in group 6; the bottom panel displays the intensity profiles across the vertical lps. (C) Image of the lps from groups 4 to 6 of the resolution target captured from the MR headset. (D) The upper panel shows the intensity profiles across the horizontal lps of elements 4 to 6 in group 6; the bottom panel shows the intensity profiles across the vertical lps.

### Tube-Threading Test With the MR-SLP

All participants successfully completed 2 sequential tasks (Figure 4) within a reasonable timeframe (<10 min). Their ${\overset{-}{T}}_{task1}\left(p,c\right)$ and ${\overset{-}{T}}_{thr}\left(p,c\right)$ records are summarized in Tables 2 and 3, respectively. According to the records, ${\overset{-}{T}}_{task1}\left(p,c\right)$ was typically the shortest in the direct view condition (c=4). A Shapiro-Wilk test was conducted with ${\overset{-}{T}}_{task1}\left(p,c\right)$ and ${\overset{-}{T}}_{thr}\left(p,c\right)$ for each test condition. The test outcome indicated that the assumption of a normal distribution was not applicable in this dataset. Therefore, a nonparametric method, the Kruskal-Wallis test, was used to compare ${\overset{-}{T}}_{task1}\left(p,c\right)$ and ${\overset{-}{T}}_{thr}\left(p,c\right)$ under different conditions. Please find the whole dataset in Table S1 and Table S2 in Multimedia Appendix 1.

**Figure 4.**
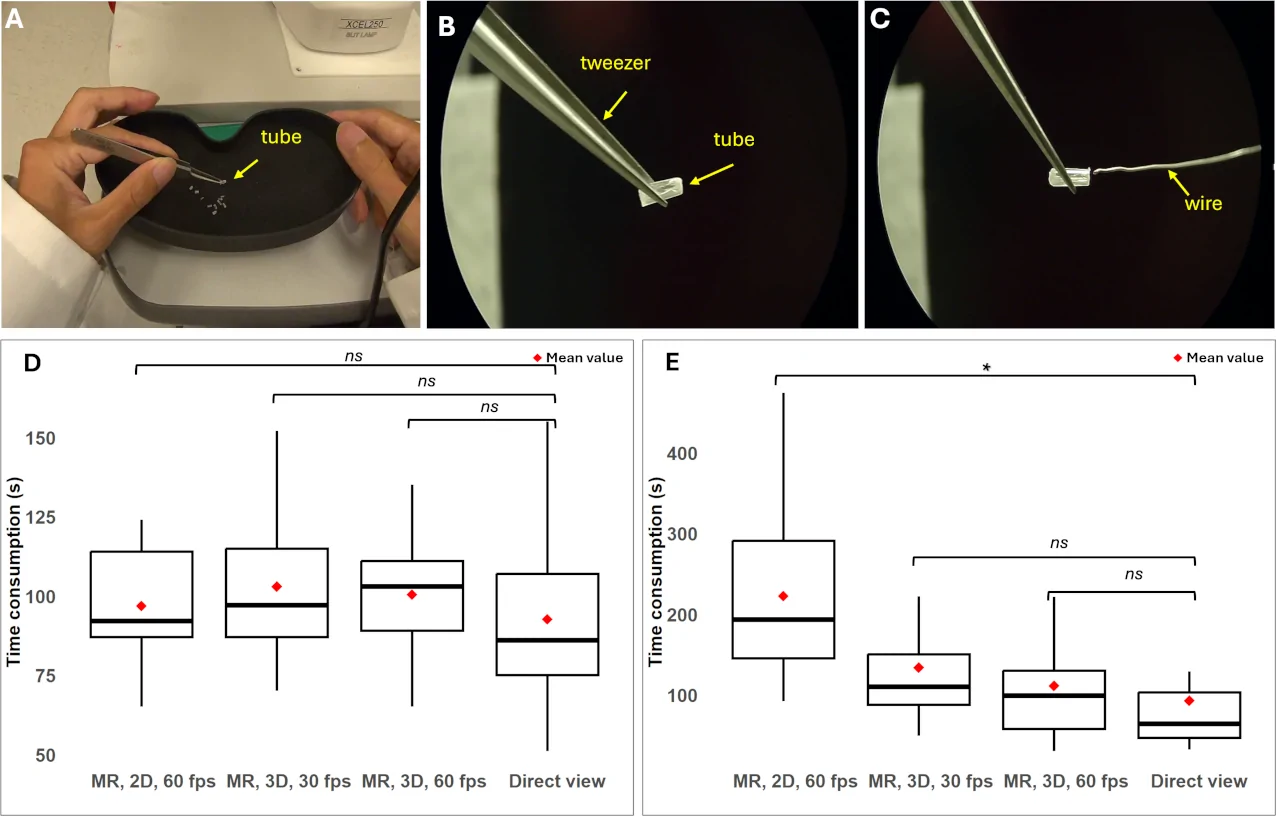
The tube-threading test and results. (A) The participant uses tweezers to grasp a tube, (B) positions the tube into the slit lamp’s field of view until it is clearly visible, and (C) threads a wire into the tube under the slit lamp’s view. (D) Box plot of $T_{task1}(p,c,i)$ across the 4 test conditions. (E) Box plot of $T_{thr}(p,c,i)$ across the 4 test conditions. Each dark dot represents an individual time record, and the red diamond symbol represents the mean value. *P<.05 between 2 conditions; fps: frames per second. MR: mixed reality; ns: no statistically significant difference.

**Table 2.** ${\overset{-}{T}}_{task1}\left(p,c\right)$ of all participants under all 4 test conditions.^a^

Participant (p)	MR^b^, 2D, 60 fps^c,d^, mean (SD)	MR, 3D, 30 fps^e^, mean (SD)	MR, 3D, 60 fps^f^, mean (SD)	Direct view^g^, mean (SD)
1	77 (9)	92 (7)	84 (18)	63 (9)
2	107 (12)	126 (15)	111 (7)	116 (29)
3	118 (4)	120 (8)	124 (22)	107 (9)
4	91 (5)	93 (8)	91 (2)	75 (5)
5	91 (11)	85 (17)	91 (19)	103 (31)

a Time was recorded in seconds, and each number represents the mean (SD) from the 5 repeats.

b MR: mixed reality.

c fps: frames per second.

d Condition ID 1.

e Condition ID 2.

f Condition ID 3.

g Condition ID 4.

**Table 3.** ${\overset{-}{T}}_{thr}\left(p,c\right)$ of all participants under all 4 test conditions.^a^

Participant (p)	MR^b^, 2D, 60 fps^c,d^, mean (SD)	MR, 3D, 30 fps^e^, mean (SD)	MR, 3D, 60 fps^f^, mean (SD)	Direct view^g^, mean (SD)
1	241 (100)	138 (77)	153 (124)	68 (29)
2	317 (119)	133 (39)	110 (20)	76 (38)
3	131 (26)	92 (14)	75 (30)	57 (18)
4	175 (56)	93 (37)	69 (25)	48 (10)
5	249 (113)	213 (120)	149 (81)	215 (139)

a Time was recorded in seconds, and each number represents the mean (SD) from the 5 repeats.

b MR: mixed reality.

c fps: frames per second.

d Condition ID 1.

e Condition ID 2.

f Condition ID 3.

g Condition ID 4.

As shown in Figure 4D, the distribution of $T_{task1}\left(p,c,i\right)$ for all 4 test conditions are similar. This observation was confirmed by the result of the Kruskal-Wallis test, indicating that there were no significant differences among ${\overset{-}{T}}_{task1}\left(p,c\right)$ for all 4 conditions (n=20; *P*=.77). According to the trend depicted in Figure 4E, the mean value of ${\overset{-}{T}}_{thr}\left(p,c\right)$ for each condition was the shortest in the direct view condition (c=4) and gradually increased from test condition 3 (MR 3D 60 frames per second [fps]) to test condition 2 (MR 3D 30 fps) and then to test condition 1 (MR 2D 60 fps). A statistically significant difference in ${\overset{-}{T}}_{thr}\left(p,c\right)$ was observed among the 4 test conditions according to the Kruskal-Wallis test (n=20; *P=*.04). Subsequent post hoc analysis using Dunn test with Bonferroni correction revealed that only ${\overset{-}{T}}_{thr}\left(p,1\right)$ and ${\overset{-}{T}}_{thr}\left(p,4\right)$ are statistically significantly different, meaning that a statistically significant difference exists only between test condition 1 (MR 2D 60 FPS) and test condition 4 (direct view; n=5; *P*=.03). This analysis showed that the performance of the threading task had no significant difference between MR headset working on 3D mode and viewing directly through the eyepieces. The results also suggested that increasing the frame rate will reduce the time taken for the threading task.

### Telemedicine Assessment

In the test, as depicted in Figure 5, the MR-SLP system’s ability to simultaneously transmit high-resolution stereoscopic video to both local and remote MR headsets was successfully demonstrated. At the local site, the operator wearing the MR headset could simultaneously see the patient through the HMD’s see-through function and view the stereoscopic examination images immersed in their real-world environment. Meanwhile, remote users connected via the internet could concentrate on the stereoscopic examination images, offering real-time expert guidance and diagnostic input, promoting effective collaboration over distances.

**Figure 5.**
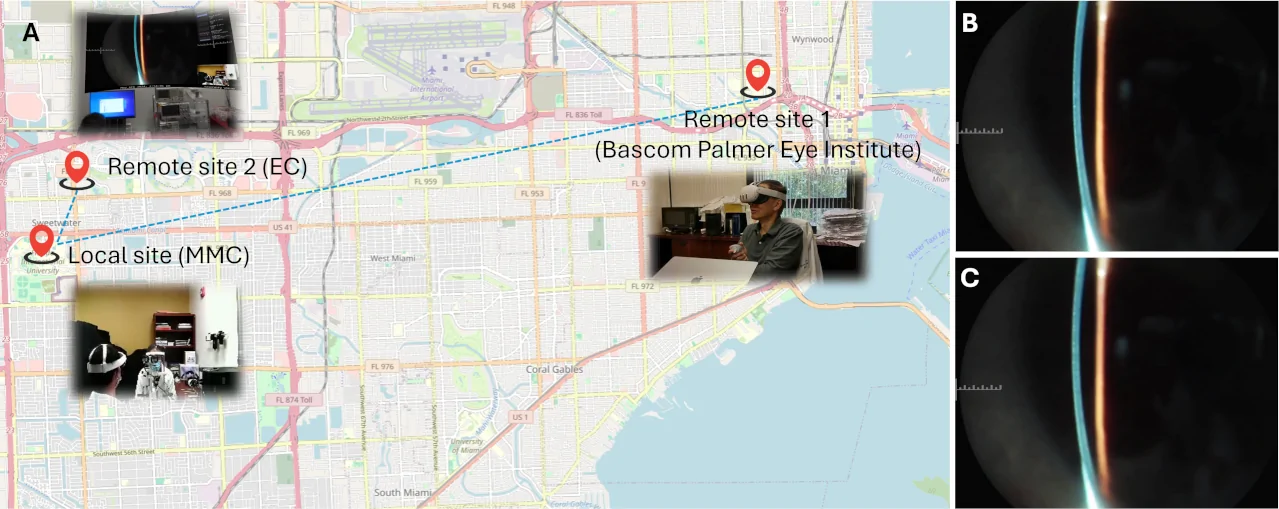
Test of mixed reality–based slit lamp (MR-SLP) for teleophthalmology. (A) The geographical relationship between the local site (Florida International University [FIU] main campus) and the remote sites (FIU Engineering Center [EC] and Bascom Palmer Eye Institute) involved in assessing image quality and user experience of the MR-SLP. (B) The images obtained directly from the camera of the MR-SLP; (C) The video frame displayed on the remote MR headset at the same time. No noticeable differences were observed between these 2 images. MMC: Modesto Maidique Campus.

During examination of the volunteer’s eye with the MR-SLP, anatomical details of the anterior segment (Figure 6) were accurately captured and displayed in real time on both local and remote MR headsets. According to the ophthalmologists participating in this trial, they reported that the image quality viewed in their MR headsets closely rivaled that observed through the slit-lamp eyepieces. The real-time video streams in this trial also maintained robust depth perception and exhibited low latency at remote sites, with an average round-trip time of less than 40 milliseconds and video jitter below 20 milliseconds. This allowed for a highly interactive and collaborative examination by the local and remote ophthalmologists. Both ophthalmologists involved in the trial stated that the MR-SLP system compared favorably to traditional slit lamp viewing and highlighted its potential advantages for clinical practice.

Fundus examination using the MR-SLP was also evaluated. Following the conventional procedure, the operator, wearing the MR headset, held an ophthalmic lens (90D; Volk Optical) between the volunteer’s eye and the slit-lamp objective to visualize the retina. An example result is shown in Figure 7. Under a nonmydriatic condition, different regions of the retina were clearly visualized in the headsets by adjusting the slit lamp and the Volk lens. Streaming of the fundus images to the local and remote headsets was processed at the background without interfering with the operator’s performance. In the test, the operator required only a brief additional period to adapt to the hand-eye coordination. When operating from alternative positions, such as an off-axis (lateral) position, maneuvering the slit lamp and Volk lens requires establishing a new hand-eye coordination and required additional practice. However, the operator could select the operating position for fundus examination without compromising overall performance.

**Figure 6.**
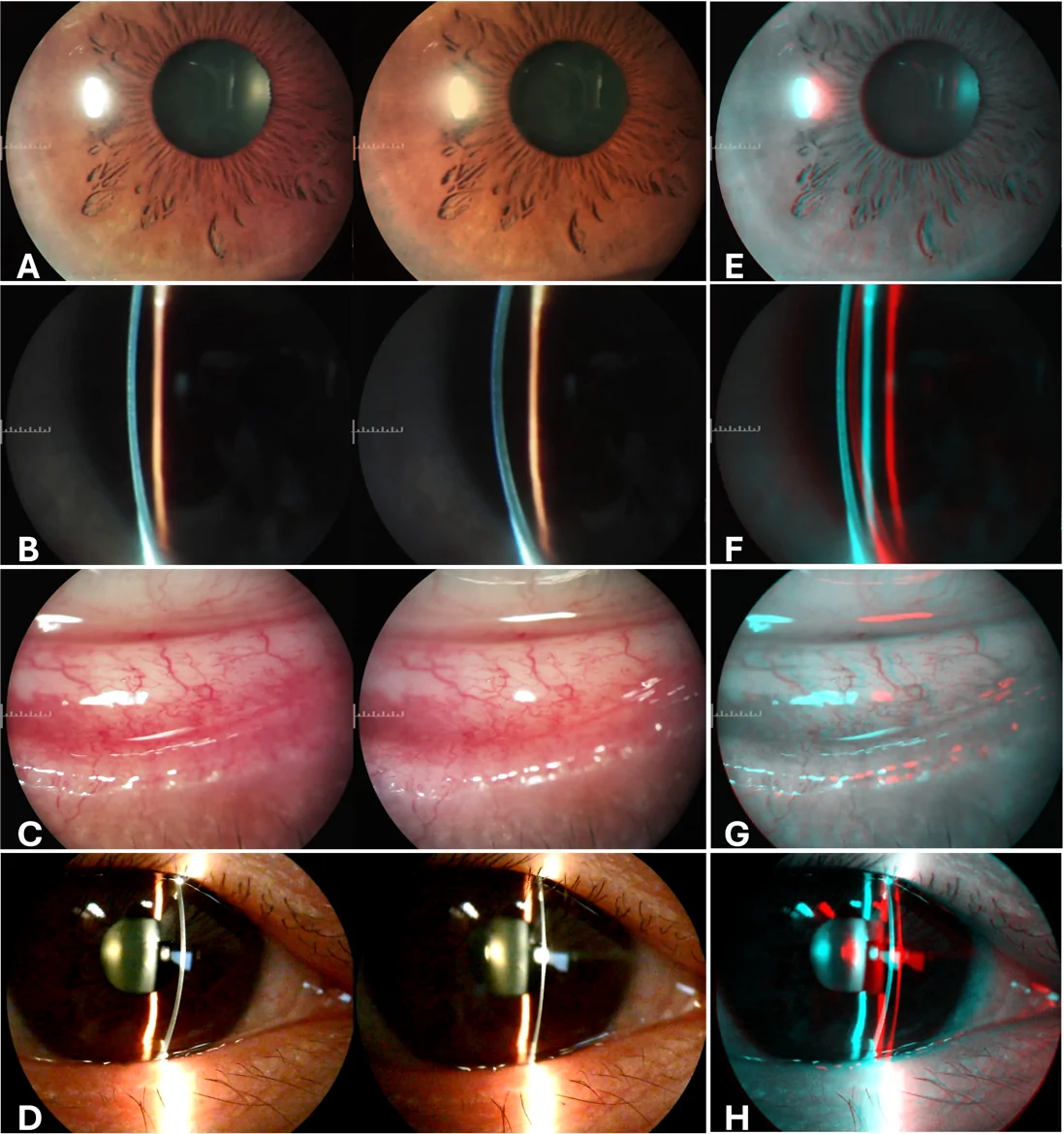
Images of the anterior segment structures captured by mixed reality–based slit lamp locally in side-by-side 3D format. (A) Iris, (B) corneal cross-section (blue stripe), (C) conjunctiva, and (D) lens. (E-H) Images in red-cyan stereoscopic format demonstrate the 3D view that can be observed using red-cyan anaglyph 3D glasses.

**Figure 7.**
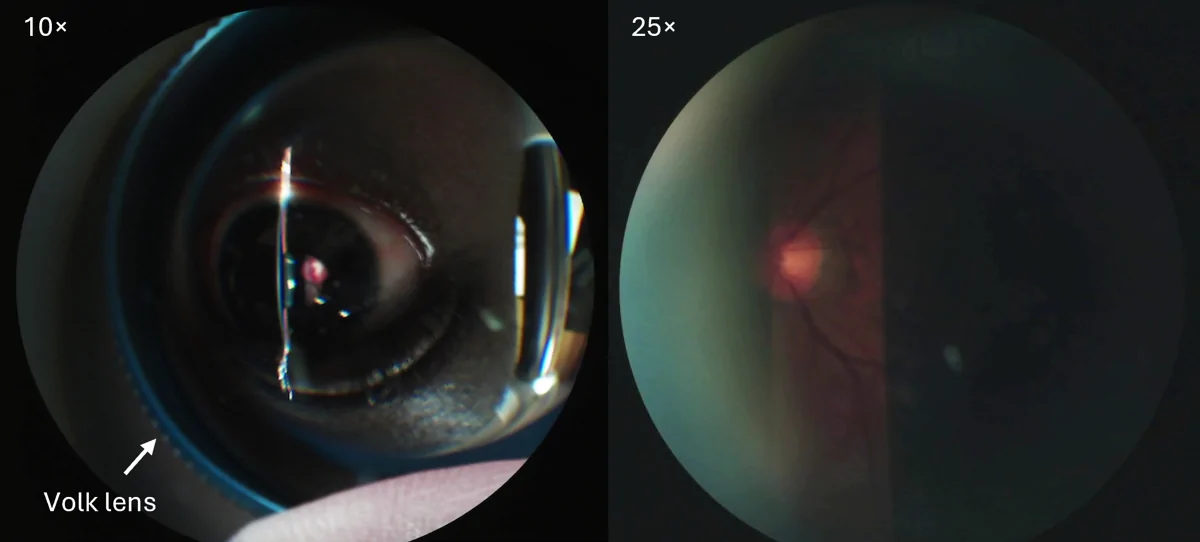
Fundus examination using the mixed reality–based slit lamp. Images were acquired through a handheld 90D Volk lens, showing retinal regions under a nonmydriatic condition at different optical magnifications.

## Discussion

### Principal Findings

We tested the performance and validated the feasibility of the MR-SLP for providing stereoscopic slit-lamp views of the eye, including the anterior segment and the fundus, in real time both locally and remotely. The MR-SLP may provide a new approach for slit-lamp examinations by eliminating the need for continuous close viewing through the eyepieces, which may offer more comfort for both the operator and the patient during examinations and more flexibility in operating the machine. Furthermore, the system enhances the efficiency of slit-lamp training by enabling multiple participants to view real-time, stereoscopic diagnostic images as if viewing them directly through the eyepieces. The remote ophthalmologist wearing the MR headset gains an immersive experience as if operating the slit lamp directly, facilitating stereoscopic teleophthalmology.

### Factors Affecting Spatial Resolution

The MR headsets used in this study have a display resolution of approximately 2K per eye, while the cameras have a maximum resolution of 4K. The measured spatial resolution was similar (102 line pairs/mm) for both the images acquired by the MR-SLP cameras and the MR device, suggesting that at the tested magnification (25×) the system’s effective resolution was limited by the slit-lamp optics. It is also possible that the MR device’s rendering algorithms enhance the perceived clarity in the region of interest, compensating partially for the lower display pixel density [15]. However, the participants in the subjective evaluation test noted a difference in perceived clarity between the MR headset and direct eyepiece view. Since this observation was based on anecdotal reports from a small group of participants (n=3), future research should implement a larger psychophysical study to statistically quantify and validate any differences in perceived image quality.

### Depth Perception, Effective Resolution, Frame Rate, and Latency

Accurate depth perception through the slit lamp is essential for precise clinical diagnosis and surgical procedures [16]. Our camera adapter and calibration algorithm allow fine-tuning of the camera position to precisely match the slit-lamp view with the digital camera view, ensuring consistent spatial mapping. The tube-threading test suggests that the MR-SLP in 3D mode may offer depth perception comparable to that of viewing through the eyepieces. Under test condition 1, participants reported that the main challenge was aligning the wire with the orifice of the tube without stereoscopic cues. A low frame rate also exacerbated the issue by creating mistiming between visual feedback and motion control, which increased the difficulty of the task. Additionally, while all participants completed a brief acclimation session, the study did not formally assess the learning curve for using the MR-SLP system. However, this preliminary test is valuable for the design of a more definitive, larger-scale validation study.

All the participants stated that the resolution target and tubes appeared clearer when viewed directly through the eyepieces. We believe this was caused by the effective resolution of the cameras used in this study during video streaming. The output frame rate was set at 60 fps only when the camera was operated at a resolution of 720p, which was limited by the camera hardware. This hardware bottleneck resulted in lower resolution of the streamed video than the slit lamp’s optics and the MR headset display could provide. This issue can be resolved by using cameras that provide 1080p or higher video resolutions at a frame rate of 60 fps, which are abundant in today’s market. The results of the tube-threading study, shown in Figure 4, also indicate that a higher frame rate led to better performance. This improvement may be attributed to the reduction of the motion blur at higher frame rates [17]. Future studies should explore how to best balance maintaining high frame rates with achieving higher resolutions, such as 1080p or 4K, for these dynamic procedural tasks. We believe that by introducing higher-quality cameras and more advanced MR headsets, such as the Apple Vision Pro, the video quality of the MR-SLP at both local and remote sites will more closely match viewing through the eyepiece of a slit lamp.

System latency is a crucial factor in real-time applications, such as telemedicine, because delays can significantly impact the local operator’s performance and diminish the quality of remote interactions. Latency can arise from network transmission, data processing, and display rendering [18]. While acceptable latency thresholds for demanding applications such as telesurgery are suggested to be below 100 to 200 milliseconds [19,20], platforms using WebRTC architecture can potentially achieve very low media transmission delays [21]. Remote tests conducted within the same city demonstrated a good performance with an average round-trip time of less than 40 milliseconds. Nevertheless, further evaluation through long-distance remote testing is needed.

### MR Headset Challenges

In the current implementation of the MR-SLP system, Meta Quest 3 is used as the MR headset for both the local and remote locations. Quest 3 offers advantages for biomedicine applications, including high-resolution displays and advanced color pass-through that enable detailed 3D visualization of anatomical structures. The see-through function and accurate spatial mapping facilitate natural interaction and situational awareness, allowing users to seamlessly integrate virtual content with the real clinical environment. Additionally, Quest 3’s onboard processing power efficiently converts side-by-side images into immersive 3D views, supporting real-time telemedicine and collaborative workflows. However, the headset’s ergonomics and comfort remain suboptimal, particularly during prolonged use, such as continuous wear for more than 60 minutes. While the display resolution is high, further improvements—such as 4K or higher per eye—would be beneficial for even greater image clarity. We are also investigating alternatives to Quest 3, such as Apple Vision Pro, or augmented reality glasses. Our system’s architecture is compatible with other extended reality platforms such as augmented reality for the local applications and virtual reality for remote physicians.

### Limitations and Future Studies

While the technique holds promise for enhancing slit-lamp examinations, teleophthalmology, and medical training, several key limitations of our study need to be acknowledged, such as small sample size for statistical analysis and subjective evaluation based on anecdotal reports.

In this study, we did not evaluate the learning curve for different operators associated with adapting to the MR-SLP view, including potential differences in hand-eye coordination during complex maneuvers. For the evaluation of user experience with the MR-SLP, feedback from participating ophthalmologists was collected through verbal reports rather than using a validated assessment scale. Although this approach provided valuable initial insights for a feasibility study, it did not allow for quantitative analysis of user satisfaction or formal measurement of interrater agreement regarding the system. Future studies should use validated questionnaires to enable a more robust evaluation of the system’s usability and acceptance in clinical settings. Additionally, the camera adapter and software implementation are cost-effective and require minimal modification, allowing for versatile integration with most slit lamps and potentially other binocular microscopes.

### Conclusions

This study demonstrated the technical feasibility of the MR-SLP in capturing and transmitting real-time, stereoscopic slit-lamp examination videos to MR headsets. Our tests showed that the system can provide visuomotor performance comparable to direct viewing, and it provided low-latency streams for effective remote collaboration.

## Supplementary material

10.2196/93513 Multimedia Appendix 1 Time required for the tube-threading tasks and picture of mixed reality–based slit lamp operation.

## Abbreviations

fps: frames per second
HMD: head-mounted display
IRB: institutional review board
MR: mixed reality
MR-SLP: mixed reality–based slit lamp
WebRTC: Web Real-Time Communication

## References

[1] Kate A, Basu S, Das T, Satgunam P (2024). Ophthalmic Diagnostics: Technology, Techniques, and Clinical Applications.

[2] Gellrich MM (2013). The Slit Lamp: Applications for Biomicroscopy and Videography.

[3] Fritz B, Paschko E, Young W (2021). Comprehensive compositional analysis of the slit lamp bacteriota. Front Cell Infect Microbiol.

[4] Shah SH, Garg AK, Patel S, Yim W, Jokerst JV, Chao DL (2021). Assessment of respiratory droplet transmission during the ophthalmic slit-lamp exam: a particle tracking analysis. Am J Ophthalmol.

[5] Dutt S, Nagarajan S, Vadivel SS (2021). Design and performance characterization of a novel, smartphone-based, portable digital slit lamp for anterior segment screening using telemedicine. Transl Vis Sci Technol.

[6] Huang Z, Yang J, Wang H, Pang CP, Chen H (2022). Comparison of digital camera real-time display with conventional teaching tube for slit lamp microscopy teaching. Curr Eye Res.

[7] Cao B, Vu CH, Keenan JD (2023). Telemedicine for cornea and external disease: a scoping review of imaging devices. Ophthalmol Ther.

[8] Schmidt TA (1975). On slit-lamp microscopy. Doc Ophthalmol.

[9] Ong CW, Tan MC, Lam M, Koh VT (2021). Applications of extended reality in ophthalmology: systematic review. J Med Internet Res.

[10] Worlikar H, Coleman S, Kelly J (2023). Mixed reality platforms in telehealth delivery: scoping review. JMIR Biomed Eng.

[11] Su GM, Lai YC, Kwasinski A, Wang H (2011). 3D video communications: challenges and opportunities. Int J Commun Syst.

[12] O’Connor AR, Birch EE, Anderson S, Draper H (2010). Relationship between binocular vision, visual acuity, and fine motor skills. Optom Vis Sci.

[13] Piano ME, O’Connor AR (2013). The effect of degrading binocular single vision on fine visuomotor skill task performance. Invest Ophthalmol Vis Sci.

[14] Deepa BM, Valarmathi A, Benita S (2019). Assessment of stereo acuity levels using random dot stereo acuity chart in college students. J Family Med Prim Care.

[15] Rao L, Argaman N, Zhuang J (2024). Display and optics architecture for Meta’s AR/VR development. IEEE Open J Immersive Disp.

[16] Bogdanova R, Boulanger P, Zheng B (2016). Depth perception of surgeons in minimally invasive surgery. Surg Innov.

[17] Li R, St George RJ, Wang X (2022). Moving towards intelligent telemedicine: computer vision measurement of human movement. Comput Biol Med.

[18] Motiwala ZY, Desai A, Bisht R, Lathkar S, Misra S, Carbin DD (2025). Telesurgery: current status and strategies for latency reduction. J Robot Surg.

[19] Wirz R, Torres LG, Swaney PJ (2015). An experimental feasibility study on robotic endonasal telesurgery. Neurosurgery.

[20] Xu S, Perez M, Yang K, Perrenot C, Felblinger J, Hubert J (2014). Determination of the latency effects on surgical performance and the acceptable latency levels in telesurgery using the dV-Trainer(®) simulator. Surg Endosc.

[21] Hamidouche W, Bariah L, Debbah M (2024). Immersive media and massive twinning: advancing toward the Metaverse. IEEE Commun Mag.

