# Supplementary material for: Mixed Reality–Based Slit Lamp for Ophthalmic Examination and Telemedicine: Technological Development and Validation Study
Source: JMIR XR Spat Comput. 2026 May 14;3:e93513. doi: 10.2196/93513 (PMC13711494; doi:10.2196/93513)
Supplement: Supplementary file 1 [file xr-v3-e93513-s001.docx]

**Table S1.** Time Required for the Tube-Threading Task1

| Participant ID | Repeats | Experimental Conditions | | | |
| --- | --- | --- | --- | --- | --- |
|  |  | HMD 2D 60fps 720p (Seconds) | HMD 30fps 720p (Seconds) | HMD 60fps 720p (Seconds) | WITHOUT HMD (Seconds) |
| 1 | 1 | 74 | 95 | 72 | 69 |
|  | 2 | 82 | 87 | 82 | 66 |
|  | 3 | 65 | 82 | 65 | 72 |
|  | 4 | 88 | 97 | 111 | 57 |
|  | 5 | 76 | 97 | 90 | 51 |
|  | SD | 8.66 | 6.76 | 17.84 | 8.74 |
|  | avg. | 77 | 91.6 | 84 | 63 |
| 2 | 1 | 102 | 112 | 115 | 164 |
|  | 2 | 120 | 152 | 107 | 121 |
|  | 3 | 117 | 126 | 121 | 105 |
|  | 4 | 103 | 122 | 105 | 89 |
|  | 5 | 92 | 118 | 107 | 100 |
|  | SD | 11.56 | 15.43 | 6.78 | 29.30 |
|  | avg. | 106.8 | 126 | 111 | 115.8 |
| 3 | 1 | 124 | 132 | 156 | 113 |
|  | 2 | 114 | 125 | 135 | 112 |
|  | 3 | 114 | 114 | 116 | 115 |
|  | 4 | 117 | 115 | 109 | 93 |
|  | 5 | 120 | 115 | 103 | 103 |
|  | SD | 4.27 | 7.98 | 21.65 | 9.18 |
|  | avg. | 117.8 | 120.2 | 123.8 | 107.2 |
| 4 | 1 | 89 | 90 | 91 | 80 |
|  | 2 | 90 | 97 | 92 | 76 |
|  | 3 | 90 | 91 | 89 | 77 |
|  | 4 | 88 | 82 | 93 | 75 |
|  | 5 | 100 | 103 | 90 | 66 |
|  | SD | 4.88 | 7.89 | 1.58 | 5.26 |
|  | avg. | 91.4 | 92.6 | 91 | 74.8 |
| 5 | 1 | 87 | 114 | 86 | 155 |
|  | 2 | 105 | 84 | 114 | 107 |
|  | 3 | 100 | 75 | 106 | 86 |
|  | 4 | 87 | 70 | 78 | 86 |
|  | 5 | 78 | 80 | 70 | 79 |
|  | SD | 10.92 | 17.25 | 18.63 | 31.11 |
|  | avg. | 91.4 | 84.6 | 90.8 | 102.6 |

**Table S2.** Time Required for the Tube-Threading Task2

|  |  | Experimental Conditions | | | |
| --- | --- | --- | --- | --- | --- |
| Subject No | Repeats | HMD 2D 60fps 720p (Seconds) | HMD 30fps 720p (Seconds) | HMD 60fps 720p (Seconds) | WITHOUT HMD (Seconds) |
| 1 | 1 | 456 | 311 | 400 | 181 |
|  | 2 | 312 | 206 | 220 | 140 |
|  | 3 | 356 | 304 | 286 | 141 |
|  | 4 | 266 | 161 | 141 | 107 |
|  | 5 | 200 | 165 | 138 | 85 |
|  | SD | 96.37 | 73.48 | 109.86 | 36.65 |
|  | avg. | 318 | 229.4 | 237 | 130.8 |
| 2 | 1 | 495 | 245 | 239 | 196 |
|  | 2 | 335 | 352 | 225 | 195 |
|  | 3 | 591 | 237 | 251 | 234 |
|  | 4 | 412 | 236 | 186 | 186 |
|  | 5 | 285 | 225 | 206 | 150 |
|  | SD | 122.80 | 52.47 | 25.93 | 29.95 |
|  | avg. | 423.6 | 259 | 221.4 | 192.2 |
| 3 | 1 | 271 | 232 | 263 | 202 |
|  | 2 | 259 | 235 | 197 | 165 |
|  | 3 | 206 | 196 | 161 | 165 |
|  | 4 | 235 | 191 | 216 | 140 |
|  | 5 | 273 | 205 | 158 | 150 |
|  | SD | 28.31 | 20.46 | 43.34 | 23.54 |
|  | avg. | 248.8 | 211.8 | 199 | 164.4 |
| 4 | 1 | 304 | 240 | 202 | 127 |
|  | 2 | 252 | 185 | 137 | 116 |
|  | 3 | 216 | 171 | 161 | 119 |
|  | 4 | 209 | 182 | 151 | 120 |
|  | 5 | 350 | 152 | 148 | 130 |
|  | SD | 60.09 | 32.84 | 25.09 | 5.86 |
|  | avg. | 266.2 | 186 | 159.8 | 122.4 |
| 5 | 1 | 363 | 479 | 358 | 546 |
|  | 2 | 460 | 402 | 293 | 448 |
|  | 3 | 451 | 233 | 240 | 206 |
|  | 4 | 196 | 170 | 160 | 206 |
|  | 5 | 232 | 206 | 147 | 182 |
|  | SD | 122.12 | 134.77 | 89.14 | 167.68 |
|  | avg. | 340.4 | 298 | 239.6 | 317.6 |

**Figure S1.** Local Operator Maneuvering the Slit Lamp with MR‑SLP. A local operator sat behind the eyepieces while wearing the MR headset, maintaining sufficient proximity to comfortably maneuver the slit lamp or ophthalmic lens without being constrained by the eyepieces.
